# Five-Factor Model and DSM-5 Alternative Model of Personality Disorder Profile Construction: Associations with Cognitive Ability and Clinical Symptoms

**DOI:** 10.3390/jintelligence11040071

**Published:** 2023-04-08

**Authors:** Chloe Lau, R. Michael Bagby, Bruce G. Pollock, Lena Quilty

**Affiliations:** 1 Centre for Addiction and Mental Health, Toronto, ON M6J 1H4, Canada; 2 Department of Psychiatry, Schulich Medicine and Dentistry, Western University, London, ON N6A 3K7, Canada; 3 Department of Psychology and Psychiatry, University of Toronto, Toronto, ON M5G 1X6, Canada; 4 Department of Psychiatry, University of Toronto, Toronto, ON M5G 1X6, Canada

**Keywords:** personality, class, profile, Big Five, DSM, person-centered approach

## Abstract

Although numerous studies have explored latent profiles using the Five-Factor Model (FFM) of normative personality, no studies have investigated how broad personality traits (i.e., FFM) and pathological personality traits using the alternative model of personality disorder (AMPD) may combine for latent personality profiles. The present study recruited outpatients (N = 201) who completed the Big Five Aspects Scales (BFAS), Personality Inventory for DSM-5 (PID-5), Structured Clinical Interview for DSM-IV (SCID-I/P), gambling and alcohol use measures, and the Weschler Intelligence subtests. When FFM and AMPD measures were combined, latent profile analyses revealed four profiles, Internalizing-Thought disorder, Externalizing, Average-Detached, and Adaptive. Detachment and openness to experience were the most and least essential traits for profile distinction, respectively. No associations between group membership and cognitive ability measures were found. Internalizing-Thought disorder membership was linked with a current mood and anxiety disorder diagnosis. Externalizing profile membership was associated with younger age, problematic gambling, alcohol use, and a current substance use disorder diagnosis. The four FFM–AMPD profiles overlapped with the four FFM-only and three AMPD-only profiles. Overall, the FFM–AMPD profiles appeared to have better convergent and discriminant validity with DSM-relevant psychopathology.

## 1. Introduction

There is an increasing trend to employ a person-centered methodology to illuminate the within-person organization of personality (Isler et al. 2017; Merz and Roesch 2011; Nylund-Gibson et al. 2007; Oberski 2016; Specht et al. 2014). Although traditional approaches consider the differential effects of traits separately (e.g., correlations between five factors of personality and variables of interest), a person-centered approach aims to detect specific subgroups based on unique configurations of trait scores that may then be used to predict and explain psychosocial and cognitive associations (Bhullar et al. 2017; Lanza et al. 2013; Spurk et al. 2020). Personality trait configurations are dimensional prototypes or abstractions in which some individuals are more closely matched to a specific prototype than others (Donnellan and Robins 2010). Within a latent profile, the varying mean value in a dimension is known as the “level”, whereas the “shape” corresponds to the differences in the relative position of a dimension within the framework (Gabriel et al. 2015; Spurk et al. 2020; Weller et al. 2020). For example, a profile with the same shape and different levels may indicate that individuals tend to have high, medium, or low levels of these traits without varying interindividual patterns. Notably, personality profiles may account for shared variance across personality traits and form configurations concerning other traits that predict engagement in and dynamic interactions with the social environment (Costa and McCrae 1992; Holloway et al. 2017; Merz and Roesch 2011; Wang and Hanges 2011). Although promising, more research is needed on whether personality profiles have predictive validity and also demonstrate incremental validity above individual trait information (Asendorpf 2015; Asendorpf and Denissen 2006; Donnellan and Robins 2010; Lanza and Cooper 2016).

### 1.1. Five-Factor Model of Personality

The Five-Factor Model (FFM) of personality is the most frequently employed model to investigate profiles based on personality traits. The FFM is composed of five broad dimensional domains, including neuroticism, agreeableness, extraversion, conscientiousness, and openness-to-experience, and is widely accepted as the predominant model for organizing personality traits commonly observed in the general population across a wide range of different languages and cultures (McCrae and Terracciano 2005). Given this universality, it is unsurprising that both behavioral genetic and family/twin studies have revealed these traits to be highly heritable (Jang et al. 2006). Moreover, variable-oriented factor analytic studies of the trait adjective checklist and objective personality instruments have repeatedly found the replicability of these five factors (McCrae et al. 1998; McCrae 2002). From a person-oriented variable approach, and by using these five domains as the foundational base, Yin et al. (2021) conducted a systematic review of 34 empirical studies, demonstrating that three- and four-profile solutions were the most dominant personality profiles using the FFM across different countries and cultures. Across the different studies, depending on the samples, the number of profiles extracted ranged from two to five depending on sample characteristics (Yin et al. 2021). In examining the broad range of labels provided for these profiles, Yin et al. (2021) recommended using the terms overcontroller, undercontroller, and resilient for consistency and comparability across studies. These terms align with Block and Block’s (1980) theoretical framework for ego-resilience (i.e., adaptive flexibility and resourcefulness) and ego-control (i.e., impulse control) domains (Barbaranelli 2002; Block and Block 1980; Bohane et al. 2017). 

Although the terminology used may be more consistent across extracted profiles with these labels, the characteristics of the undercontroller, overcontroller, and resilient profiles may differ based on shape and level depending on sample characteristics, such as culture, age, and gender (Bohane et al. 2017; Yin et al. 2021). Consistent with empirical findings on resilience (e.g., Oshio et al. 2018), the features of a resilient profile were most replicable with the lowest scores in the neuroticism domain and highest scores in the extraversion, conscientiousness, agreeableness, and openness to experience domains compared to other profiles that emerge in each sample (Yin et al. 2021). The resilient profile is associated with positive attributes (e.g., better financial wealth, likeability, sociability, leadership qualities) and well-being (e.g., work and life satisfaction, psychosocial adjustment; Exley et al. 2022; Isler et al. 2017; Udayar et al. 2020). These results suggest that the combination of high scores in all commonly positively regarded personality characteristics combined with low neuroticism may be related to the shared variance of positive attributes across the FFM (i.e., representing socially effective behaviour and/or social desirability), analogous to the positive characteristics linked with the General Factor of Personality (GFP) located at the top of the hierarchical structure of personality (Do and Minbashian 2020; Dunkel et al. 2014; Erdle et al. 2010; Musek 2007; van der Linden et al. 2010a) reported in factor analytic studies. 

Within this model, the term undercontrollers was used to depict low ego-resiliency and low ego control, which was represented by low agreeableness and conscientiousness within the FFM (Bohane et al. 2017; Yin et al. 2021). Overcontrollers were identified with their low ego-resiliency and high ego-control, which was represented by high neuroticism combined with low extraversion and openness to experience (Bohane et al. 2017). In contrast to the resilient profile, children who were once categorized as undercontrollers or overcontrollers were more likely than resilient children to be diagnosed with a psychiatric disorder, experience interpersonal difficulties, and endorse significant psychopathology (Caspi 2000; Caspi et al. 1996; Caspi et al. 2003; Causadias et al. 2012; Klimstra et al. 2010; Newman et al. 1997; Slutske et al. 2012). Based on these attributes, undercontrollers are at greater risk for high levels of aggression, future violent offense, externalizing psychopathology, and substance use, whereas overcontrollers are at greater risk of internalizing symptoms and emotional volatility (Bohane et al. 2017; Caspi et al. 1996; Denissen et al. 2008; Exley et al. 2022; Klimstra et al. 2010; Hart et al. 2003; Robins et al. 1996; Thompson-Brenner and Westen 2005; Westen and Harnden-Fischer 2001; Wildes et al. 2011; Yin et al. 2021). Within clinical samples, the prevalence of overcontrollers ranges from 9% to 43% (M = 28%), while undercontrollers range from 31% to 49% (M = 40%; Bohane et al. 2017). Within the FFM framework, the constructs underlying overcontrol and undercontrol may relate to the “Big Two” of Plasticity and Stability, respectively (DeYoung et al. 2002; Digman 1997; DeYoung 2010; van der Linden et al. 2010a). Overcontrol and low plasticity (i.e., shared variance between extraversion and openness to experience) may predispose an individual to avoid engaging with novel social and/or intellectual stimuli, thus impairing psychosocial and vocational functioning (Digman 1997; DeYoung 2010; van der Linden et al. 2010a, 2010b). Symptomology related to undercontrol may be related to low stability (i.e., shared variance between neuroticism, conscientiousness, and agreeableness), as both undercontrol and low stability are associated with difficulties with impulse control and maintaining effortful goal-oriented behaviour. Yet, in addition to the resilient, overcontroller, and undercontroller profiles, Isler et al. (2017) argued that a four-profile solution may provide greater consistency and predictability than the three-profile model. Such profiles have various labels (e.g., Average, Ordinary, Brittle) with different characteristics, which creates difficulties in generalization (Yin et al. 2021). Although results are mixed regarding the suitability and features related to a fourth and/or fifth profile, the overcontroller, undercontroller, and resilient profiles have emerged across different genders, cultures, methodological techniques, and assessment instruments (Bohane et al. 2017).

### 1.2. Alternative Model of Personality Disorder 

Despite the fact that the FFM has delivered thematically meaningful profile prototypes, one limitation is that it is designed to capture “normal range” traits and not pathological traits (extreme scores on the dimensional poles of each of the five factors may be indicative of psychopathology, however). Daljeet et al. (2017) have argued that a person-centered approach incorporating broad personality characteristics and maladaptive personality traits may be beneficial in identifying additional variance not expressed by the FFM. A normal-range personality is closely linked with psychopathology (Kotov et al. 2010; Krueger and Tackett 2003; Trull and Widiger 2022; Widiger and Mullins-Sweatt 2009). Similar models have been developed using pathological traits; however, most of the research regarding normative and pathological personality is conducted separately (Uliaszek et al. 2015). The assessment of dimensional personality traits is measured using the Alternative Model of Personality Disorders (AMPD) presented in Section III of the Diagnostic and Statistical Manual of Mental Disorders (5th ed. [DSM–5]; American Psychiatric Association 2013; Watters et al. 2019; Weekers et al. 2021). The AMPD and FFM are both composed of five higher-order domain traits, four from each which are largely conceptually similar (e.g., neuroticism and negative affectivity, agreeableness and antagonism, extraversion and detachment, and conscientiousness and disinhibition). While the AMPD includes a psychoticism trait domain, the FFM does not. Conversely, while the FFM includes an openness-to-experience domain, this domain does not appear in the AMPD. Although the FFM traits are dimensionally bipolar, the AMPD is unipolar in that only high scores represent pathological personality. These differences reflect the conceptual nature of these two models (Chmielewski et al. 2014; Gore and Widiger 2013). Although there has been extensive research on the dimensional measurement of the AMPD over the last decade, few studies have conducted latent class or latent profile analyses with the AMPD traits (e.g., Hanegraaf et al. 2022; Gamache et al. 2021). In a community MTurk sample, Hanegraaf et al. (2022) used latent class analysis to identify four classes using AMPD traits that showed hypothesized correlations with criterion validity variables (e.g., self-concept, drug use, depressive symptoms), including high psychopathology (i.e., highest endorsement of all five trait domains compared to other classes), low psychopathology (i.e., lowest endorsement of all five trait domains than other classes), and two moderate psychopathology groups. The first moderate psychopathology group was labeled internalizing, given its higher detachment symptoms than the other moderate group. The other moderate psychopathology was labeled externalizing given its higher negative affectivity, antagonism, disinhibition, and psychoticism than the other moderate groups (Hanegraaf et al. 2022). However, given that the number and types of profiles largely depend on the nature of the sample, it is crucial to replicate the number, profile shape (i.e., means across traits within profiles), the variance of dimensions, and size of groups with the AMPD, particularly within clinical samples where such profiles may demonstrate clinical relevance and utility.

### 1.3. Study Objectives 

The objectives of our study are three-fold. Our first objective is to (1a) attempt to replicate in an adult clinical sample separate FFM and AMPD latent profile types extracted in previous research that used online crowd-sourcing community and university samples, and (1b) to distinguish whether the extracted profile types, in turn, are uniquely associated with different external criteria. As Donnellan and Robins (2010) argued, the nature of “convenience” community samples, as well as university student samples, are less likely to produce overcontroller and undercontroller profiles with significant levels of psychopathology. Moreover, we hypothesize that similar profiles will emerge from previous findings and that a clinical sample would show more extreme variants of AMPD scores and perhaps differential latent profiles compared to community samples. The replication of these results in clinical samples with a broader range of psychiatric symptoms is needed, including the evaluation of overlap with other constructs (e.g., mental health disorders, cognitive ability) from a subjective, clinician-rated, and performance-based standpoint. The present study reveals both self-report (i.e., self-report version of the AUDIT) and clinician-rated (i.e., clinician-rated SCID-I/P) problematic substance use. Previous findings have reported criterion validity of profiles based solely on self-report measures, which are subjected to the common method bias. It is unclear whether participants across different groups may differ across clinician-rated diagnoses or cognitive ability. Previous findings indicated cognitive ability and personality traits are linked (Rammstedt et al. 2016). Specifically, cognitive ability is positively associated with emotional stability and openness, while negative associations were found between conscientiousness and cognitive ability (Rammstedt et al. 2016). Given that the profiles reveal an amalgamation of differential personality traits, it becomes imperative to reveal whether cognitive ability may be associated with specific profile memberships.

Our second objective is to extract latent profile types from a combined pool of FFM and AMPD traits and compare them with profiles extracted from FFM-only and AMPD traits. Both Donnellan and Robins (2010) and Bohane et al. (2017) have argued that broad trait pools are more likely to contain a sufficiently wide range of personality constructs that would permit the adequate capture of the overcontrol and undercontrolled profile types. We hypothesize that combining the “normal range” FFM traits and pathological traits of the AMPD traits afford the expansion domain of traits. Of note, the AMPD consists of Criterion A (i.e., severity of personality dysfunction) and Criterion B (i.e., endorsement of one or more pathological domains and traits). This study does not study AMPD Criterion A but, instead, focuses on measuring the severity of one or more pathological traits within profiles. Gamache et al. (2021) formulated profiles based on Criterion A and Criterion B to form profiles based on borderline pathology, indicating that the addition of Criterion A may yield more fine-grained profiles (i.e., with differences in both levels and shape). Notably, the current profiles do not assess the severity of personality dysfunction but, instead, capture the endorsement of specific traits and attributes.

Although the FFM and AMPD trait pools manifest some conceptual overlap, item content also differs. The complementary latent traits of interest in this investigation are “quasi-traits”, in which a unipolar dimension measures the presence or absence of a trait (e.g., antagonistic vs. non-antagonistic, agreeableness vs. not agreeable; Reise and Waller 2009). Although the commonly positively regarded personality traits may be maladaptive on one end, the incorporation of maladaptive personality traits may account for additional variance (Williams and Simms 2018). The assessment of the bipolar trait, in which both extremes at opposite ends of a spectrum may identify meaningful variants of a construct (e.g., antagonistic vs. agreeable), may provide more information clinically. Thus, measuring both extreme ends of the latent continuum ensures a more comprehensive and precise measurement across the bipolar spectrum in a person-centered approach, which enables more discriminant measurement of personality strengths and pathology (Reise and Waller 2009).

Our third and final objective is to distinguish whether these profiles from FFM-only (i.e., includes only broad personality traits), AMPD-only (i.e., includes only PID-5 traits), and FFM–AMPD combined profiles are linked with cognitive ability, problematic alcohol use, problematic gambling, and DSM-5 diagnoses as rated by a clinician. Most adult studies have relied on self-report associations and evidence on conceptual overlaps between these profiles with clinical observations (e.g., DSM diagnoses, problematic substance use) remains limited. The clinical sample and multimodal assessment add new and potentially theoretically meaningful information about the criterion validity of FFM–AMPD profiles.

## 2. Materials and Methods

### 2.1. Sample and Procedure

The recruitment for the current study employed a research registry at a large academic Canadian mental health and addiction facility. Participants had attended appointments in various outpatient treatment and assessment clinics with different focuses (e.g., pharmacotherapy and psychotherapy) within the past 12 months. A total of 871 potential study participants from the registry were contacted and provided an overview of the study. Of these, 354 completed a telephone screen for eligibility. All participants were provided with information that partaking in this study was voluntary and that any information or data collected in the course of the study would be anonymized. Participants were also made aware of the purpose and duration of the study and that they could withdraw from the study at any time. Inclusion criteria for the study were specified as the presence of clinically significant psychiatric symptoms and active engagement within the hospital in the last year. Exclusion criteria included a current or lifetime psychotic disorder, severe homicidal or suicidal ideation, and intoxication or withdrawal.

Of those who completed the telephone screening, 201 participants (50% women), aged 18 to 87 years old (M = 39.66, SD = 13.76), attended the research clinic and completed the study. Participants identified with the following racial/ethnic backgrounds: Asian/Pacific Islander (9%), Black (3%), European White (76%), First Nations (4%), Latin American (5%), and other (e.g., multiracial; 5%). After participants provided verbal and written consent, they completed two assessment sessions, which included interviewing, the completion of questionnaires, and the administration of cognitive measures. Upon the completion of the study, participants were debriefed and provided monetary compensation for their time and efforts. The hospital Research Ethics Board approved the study procedures. 

### 2.2. Measures of Personality

Five-Factor Model of Normative Personality. The Five-Factor Model of Personality (FFM) was measured with the Big Five Aspects Scales (BFAS; DeYoung et al. 2007) and is comprised of 100 self-report items that related to five broad personality factors. A total of 10 lower-order aspects may be extracted from the five domains: openness to experience (intellect, openness), conscientiousness (industriousness, orderliness), extraversion (enthusiasm, assertiveness), agreeableness (compassion, politeness), and neuroticism (volatility, withdrawal). Respondents rate each statement on a five-point Likert-type scale ranging from 1 (very inaccurate) to 5 (very accurate). Previous findings have confirmed the reliability (internal) and validity of this measure (see, e.g., DeYoung et al. 2007). For this measure in this study, reliabilities for the five factors using McDonald’s ω ranged from 0.84 to 0.91 (see Table 1 for all values). 

Alternative Model of Personality Disorders (AMPD). The Personality Inventory for DSM-5 (PID-5; Krueger et al. 2012) was designed to assess maladaptive traits of the AMPD. It consists of 220 self-report items. The PID-5 measures 5 higher-order broad personality domains (i.e., Negative Affect, Detachment, Antagonism, Disinhibition, Psychoticism) and 25 lower-order personality facets. Items are rated on a four-point, Likert scale ranging from “0” (very false or often false) to “3” (very true or often true). Reliabilities for the five domain scales McDonald’s ω range from 0.81 to 0.88. Previous research supported the solid internal consistency, structural validity, and concurrent validity for each subscale (Al-Dajani et al. 2016; Krueger and Markon 2014; Hopwood and Sellbom 2013; Quilty et al. 2013). Miller et al. (2022) provided normative data for representative, community, and clinical samples, and demonstrated substantive differentiation between these groups. The means of the current sample correspond to that of the clinical sample (Miller et al. 2022).

### 2.3. External Validity Measures

Diagnostic Assessment. The Structured Clinical Interview for DSM-IV (SCID-I/P; First and Gibbon 2004) was provided as a semi-structured clinical interview designed to assess the presence of major mental disorders in the DSM-IV diagnosis. All SCID-I/P assessments were conducted by registered clinical psychologists (LCQ and RMB). SCID-I/P diagnoses of any mood disorders, any anxiety disorder, and any substance use disorder were categorized as current or lifetime diagnoses. Any DSM-5 mood disorder was categorized as a major depressive disorder, dysthymic disorder, or depressive disorder not otherwise specified. Of the 201 participants who consented and met the study inclusion and exclusion requirement, 145 (72.5%) met criteria for a lifetime mood disorder and 66 (33%) met the criteria for a current mood disorder; 109 (53%) met the criteria for a lifetime substance use disorder, and 38 (18%) met criteria for a current substance use disorder. An anxiety disorder is categorized as any DSM panic disorder, agoraphobia without panic disorder, social anxiety disorder, specific phobia, obsessive compulsive disorder, post-traumatic stress disorder, generalized anxiety disorder, or anxiety disorder not otherwise specified. In the present sample, 98 (49%) met criteria for a lifetime anxiety disorder, and 64 (32%) met the criteria for a current anxiety disorder. 

Alcohol Use and Dependence. The Alcohol Use Disorders Identification Test (AUDIT) is a reliable and valid measure of risky and harmful drinking patterns. This ten-item measure yields scores from 0 to 40 that estimate alcohol use frequency, the quantity of drinking, dependence or tolerance symptoms, and other problems associated with alcohol use (Babor et al. 2001; Saunders et al. 1993). A score of five or higher connotes problematic alcohol use and hazardous drinking (Nadkarni et al. 2019). For the current sample, the posterior mean for Bayesian McDonald’s ω is 0.90 with 95% Credible Intervals (CIs) ranging from 0.88 to 0.92. Previous research demonstrated strong internal consistency, test–retest reliability, structural validity, and criterion validity (de Meneses-Gaya et al. 2009).

Problematic Gambling. The Problem Gambling Severity Index (PGSI) is a reliable and valid nine-item self-report measure that estimates the frequency and severity of problem gambling over the past year for the general population (Ferris and Wynne 2001; Holtgraves 2009). The total score ranges from 0 to 27, as each item was rated on a 0–3 scale, with higher scores indicating increasingly problematic gambling behavior. Total scores are evaluated as follows: 0 reflects no problems, 1 to 2 represents low risk, 3 to 7 represents a moderate risk with some negative consequences, and 8+ represents problematic gambling with significant negative consequences (Ferris and Wynne 2001; Holtgraves 2009). For this study, a cut-off value of 3 was indicated (Currie et al. 2010). The Bayesian McDonald’s ω posterior mean equals 0.90, with the probability that McDonald’s ω lies between 0.88 and 0.92 at 95%. The PGSI has strong psychometric properties and discriminant abilities to differentiate the general population from problematic gamblers (McMillen and Wenzel 2006; Miller et al. 2013). 

Verbal Comprehension. The Wechsler Adult Intelligence Scale–III (WAIS-III) Vocabulary Subtest is a comprehensive assessment of knowledge in vocabulary, level of comprehension of different words, ability to express vocabulary, and verbal concept formation (Wechsler 1997). Using standardized procedures according to the manual, the WAIS-III was administered and scored with raw scores converted to age-corrected scaled scores based on standard age group norms (Jeyakumar et al. 2004; Wechsler 1997). 

Matrix Reasoning. The WAIS-III Matrix Reasoning subtest is a comprehensive test that assesses visuospatial reasoning. Raw scores were converted to age-corrected scaled scores (Wechsler 1997).

### 2.4. Statistical Analyses

Table 1 shows the means, standard deviations, bivariate Bayesian Pearson’s rho correlations, and Bayesian single-test McDonald’s ω for the BFAS and PID-5 broad personality factors (Pfadt et al. 2022). Three separate latent profile analyses were conducted with (1) FFM-only (i.e., neuroticism, openness-to-experience, extraversion, conscientiousness, agreeableness), (2) AMPD-only (i.e., negative affectivity, detachment, antagonism, psychoticism, disinhibition), and (3) FFM–AMPD combined (i.e., all 10 factors of normative and pathological personality). Personality trait domain scales from the BFAS and PID-5 were standardized into z-scores prior to estimation to enhance the interpretability of profiles. Means and variances of the profile indicators were freely estimated (Diallo et al. 2016). The expectation-maximization (EM) algorithm was employed to gather the maximum likelihood estimates for the parameters. Low values of sample size adjusted BIC (SABIC), Bayesian Information Criterion (BIC), and Akaike Information Criterion (AIC), as well as significance of the Bootstrapped Likelihood Ratio Test (BLRT) used for selecting the best fitting solution (Nylund-Gibson et al. 2007; Spurk et al. 2020; Weller et al. 2020). The entropy index was employed to assess the profiles’ discriminant abilities, with a cut-off of >0.80 suggesting acceptable certainty (Celeux and Soromenho 1996; Wang et al. 2017). Only profiles with >5% membership of the total sample were extracted to avoid over-extraction and spurious profiles (Spurk et al. 2020; Weller et al. 2020). The optimal model was the most parsimonious with fit, class size, and interpretability based on theoretical framework (Spurk et al. 2020; Weller et al. 2020). 

Age differences were computed using Bayesian ANOVA, and gender differences were compared using frequentist chi-square and Bayesian multinomial contingency tables (Wetzels et al. 2012; Wagenmakers et al. 2018). Yin et al. (2021) defined the most important variable as the dimension that plays the most important role in differentiating profiles and providing discernable differences in mean values. With this definition, ANCOVAs were applied to each variable that was used to construct the profile to determine the effect size (η^2^ and ηp^2^) and Vovk-Sellke Maximum p-Ratio (VS-MPR) of each variable in differentiating between profiles. The most and least essential variables were defined as the variable with the largest and lowest values, respectively, of VS-MPR, ηp^2^, and η^2^. 

The AUDIT was normally distributed (skewness = 1.54; kurtosis = 1.72) but the scores on the PGSI (skewness = 3.51; kurtosis = 15.97) were not normally distributed. A log10 transformation with an added value of 0.50 to the PGSI total scale score prior to transformation was subsequently used to account for non-normality and values of zero (Mercer and Eastwood 2010). With criticisms of log transformations and implications that it may not reflect the original data, chi-square tests with untransformed variables were also employed to categorize respondents with or without self-reported difficulties with problematic gambling (Feng et al. 2014). 

Once extracted, these profiles were compared across the numerous criterion variables, including DSM diagnoses (i.e., anxiety, substance use, and mood disorders), problematic alcohol use and gambling, and cognitive ability (i.e., verbal comprehension and matrix reasoning). Mean differences of theoretically relevant symptoms were conducted across established profile membership using the Bayesian ANCOVA methodology (Rouder et al. 2012). Considering the broad age range in the current sample and potential gender differences in clinical symptoms and cognitive ability, age, and gender were controlled as covariates. Bayesian ANCOVA incorporates two model comparisons between the null model that contains the grand mean and the criterion validity model containing the profile membership of interest. A model including profile, gender, and age was compared to the model containing gender and age (Wagenmakers et al. 2018; van den Bergh et al. 2020). Default multivariate Cauchy priors were not changed (fixed effects Cauchy prior scale parameter for fixed effects = 0.50 with Cauchy prior scale parameter for covariates = 0.354). Prior odds were adjusted for multiple comparisons (Westfall et al. 1997). Bayes factors may be interpreted as follows: 1 as no evidence, 1–3 as anecdotal evidence for H1, 3–10 as moderate evidence for H1, 10–30 as solid evidence for H1, 30–100 as robust evidence for H1, and > 100 as extreme evidence for H1 (Jeffreys 1961; Lee and Wagenmakers 2014; Stefan et al. 2019). 

Frequentist chi-square tests of independence and Bayesian multinomial tables were used to determine whether profile membership was associated with DSM diagnoses. Standardized residuals may be interpreted with >1.96 (i.e., 25th and 97.5th percentiles assuming a true null), indicating that the number of cases in that cell is significantly larger than expected if the null hypothesis were true at *p* = 0.05. The standardized residuals are computed with the following equation: (observed − expected)/sqrt(expected × [1 − row marginal proportion] × [1 − column marginal proportion]). Vovk-Sellke Maximum p-Ratio is also reported as the maximum possible odds in favor of the alternative hypothesis over the null hypothesis which equates to 1/(−e p log[p]) for *p* ≤ 0.37 (Sellke et al. 2001). 

Descriptive and Bayesian statistics were conducted on JASP 0.16.4. Jamovi version 2.3 was employed to conduct latent profile analyses with the snowRMM add-on module (Rosenberg et al. 2019; Şahin and Aybek 2019; The Jamovi Project 2022).

## 3. Results

### 3.1. Five Factor Model-Only (FFM-Only) Profiles

In a latent profile analysis of the FFM-Only domains, a four-profile model yielded the best fit to the data as suggested by theoretical considerations and profile interpretability shown in Table 2. One profile for the five-profile solution yielded a profile with <5% of participants. Close examination of the five-profile solution suggests that a fifth profile did not enhance interpretability or create a distinct profile with varying level and shape compared to others. 

**Figure 1:**
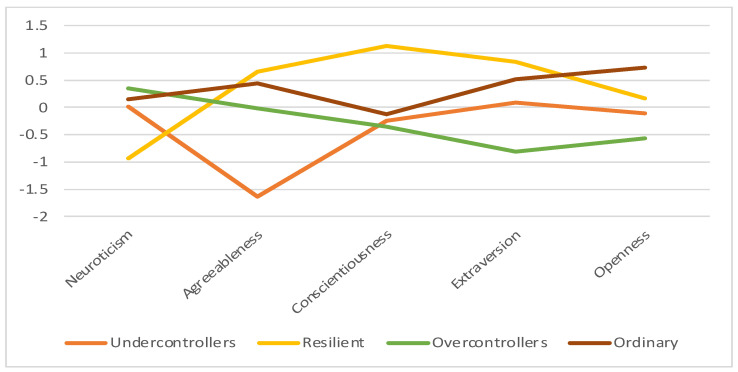
Profile plot for Five Factors in FFM-Only Profiles. Note. Standardized mean scores (M = 0, SD = 1) of the Five-Factor Model of Personality across four profiles.

The FFM-only four-profile solution was named descriptively consistent with profiles, as previously labeled by Yin et al. (2021) as (1) Undercontroller, (2) Resilient, (3) Overcontroller, and (4) Ordinary (Figure 1). The Undercontroller profile members (14.4% of participants; 55.6% male, 44.4% female) were between the ages of 20 and 56 (M = 33.54, SD = 12.09). This group was labeled following the lowest scores on agreeableness, low scores on conscientiousness, and mid-ranged scores on neuroticism, extraversion, and openness to experience compared to other profiles. The Resilient profile members (18.5% of participants; 41.7% male, 58.3% female) were between the ages of 22 and 71 (M = 43.58, SD = 13.39) and demonstrated the lowest scores in neuroticism, highest scores in agreeableness, conscientiousness, and extraversion, and mid-ranged scores in openness to experience compared to other profiles. The Overcontrollers group (39.5% of participants; 57.1% male, 42.9% female) between the ages of 18 to 87 (M = 42.12, SD = 14.28) consists of high scores on neuroticism and low scores in conscientiousness, extraversion, and openness to experience. This profile is also characterized by mid-ranged agreeableness. The Ordinary profile (27.7% of respondents; 49.5% male; 50.5% females) were between ages 18 to 62 (M = 36.17, SD = 11.99) and exhibited mid-ranged neuroticism, agreeableness, conscientiousness, and extraversion. This term was amongst those most commonly used when profiles demonstrate mid-range responses on traits (Yin et al. 2021). This profile has the highest openness to experience score compared to other profiles. The most critical variable in determining profiles was agreeableness, as evinced with the largest effect size and VS-MPR (ηp^2^ = 0.58; VS-MPR = 1.31 × 1032), reflecting more significant differences between profiles. The least essential variable is openness to experience, as apparent with the smallest effect size and VS-MPR (ηp^2^ = 0.33; VS-MPR = 1.48 × 1013). Details are found in Appendix A. 

Demographic effects on profile membership were assessed and no gender differences were found (χ^2^[3, N = 201] = 4.74, *p* = 0.19, BF_10_ Independent Multinomial = 0.14). Age predicted profile membership (P[M] = 0.50, P[M|data] = 0.93, BF_M_ = 14.14, error % = 0.02). With posterior odds corrected for multiple testing with prior odds set as 0.41 (Westfall et al. 1997), there was strong evidence that the Undercontroller profile (M = 33.54, SD = 12.09) was younger than the Resilient profile (M = 43.58, SD = 13.39; Posterior Odds = 5.31; BF_10_ = 12.83, error% < 0.001). There was moderate evidence that the Undercontroller profile members were younger than Overcontroller profile members (M = 42.12, SD = 14.28; Posterior Odds = 2.93, BF_10_ = 7.08, errors% < 0.001), Ordinary profile (M = 36.17, SD = 11.99) was younger than the Resilient profile (Posterior Odds = 2.33, BF_10_ = 5.63, error% < 0.001), and the Ordinary profile was younger than the Overcontroller profile (Posterior Odds = 1.30, BF_10_ = 3.14, error% < 0.01). No evidence was found for other age differences between profiles. 

### 3.2. Associations of FFM Only Profiles with Clinical Symptoms and Cognitive Ability Measures

When accounting for the error variance and the effects of gender and age, profile membership was associated with problematic gambling. In particular, the model with profile, gender, and age (P[M] = 0.13, P[M|Data] = 0.59, BF_M_ = 9.97, BF_10_ = 422.62, error% = 0.88) compared to the gender and age model (P[M] = 0.13, P[M|data] = 0.05, BF_M_ = 0.34, BF_10_ = 33.00, error% < 0.01) was a better fit for the data. The model with profile membership with covariates was preferred to the covariates only model by a factor of 13 (422.62/33 ≃ 12.80). Post hoc comparisons suggested that the Undercontroller profile (M = 3.64; SD = 5.90) was more likely to engage in problematic gambling than the Resilient (M = 1.11, SD = 3.65; Posterior Odds = 82.81, BF_10,U_ = 199.91, error% < 0.001) and Ordinary profiles (M = 1.00, SD = 2.35; Posterior Odds = 19.95, BF_10,U_ = 48.17, error% < 0.001). When using established cut-offs (PGSI ≥ 3) for problematic gambling, a Chi-Square Test of Independence showed a significant relationship between profile membership and problematic gambling with medium effect sizes, χ^2^(3, N = 199) = 9.79, *p* < 0.05, VS-MPR = 4.62, Cramer’s V = 0.22, BF_10_ = 0.45. The number of individuals who engaged in problematic gambling was greater than expected in the Undercontroller profile (z = 2.72).

FFM profile membership did not appear to predict problematic alcohol use, matrix reasoning, or verbal abilities. When gender, age, and profile membership were entered to predict problematic alcohol use, the age-only model (P[M] = 0.13, P[M|Data] = 0.47, BF_M_ = 6.14, BF_10_ = 2.04, error% < 0.01) outperformed other models containing profile membership. Chi-square tests were consistent with these findings, with no association between profile membership and problematic alcohol use (AUDIT ≥ 5), χ^2^[3, N = 199] = 5.21, *p* = 0.16, BF_10_ = 0.18). For matrix reasoning, there was no evidence that the profiles model (P[M] = 0.50, P[M|Data] = 0.45, BF_M_ = 0.83, BF_10_ = 0.83, error% < 0.001) is a better fit than the null model (P[M] = 0.50, P[M|Data] = 0.55, BF_M_ = 1.21, BF_10_ = 1.00). Similarly, there was no evidence that the profiles model (P[M] = 0.50, P[M|Data] = 0.48, BF_M_ = 0.91, BF_10_ = 0.91, error% < 0.001) outperforms the null model (P[M] = 0.50, P[M|Data] = 0.52, BF_M_ = 1.10, BF_10_ = 1.00) for verbal reasoning.

Table 3 shows counts and standardized residuals of profile membership and diagnosis. There was no association between lifetime anxiety disorder diagnosis and profile membership (χ^2^[3, N = 200] = 0.45, *p* = 0.93, BF_10_ Independent Multinomial = 0.02). However, profile membership was significantly associated with current anxiety disorder (χ^2^[3, N = 200] = 7.90, *p* = 0.05, BF_10_ Independent Multinomial = 0.82). The Resilient group was less likely than expected to meet criteria for a current anxiety disorder (z = −2.28) and the Overcontroller group was more likely to meet criteria for a current anxiety disorder (z = 2.39). No significant relationships were found between profiles membership and lifetime substance use disorder (χ^2^[3, N = 201] = 0.35, *p* = 0.95, BF_10_ Independent Multinomial = 0.01) and current substance use disorder (χ^2^[3, N = 199] = 5.95, *p* = 0.11, BF_10_ Independent Multinomial = 0.12). No significant associations were found between profile membership and lifetime mood disorder (χ^2^[3, N = 200] = 2.97, *p* = 0.40, BF_10_ Independent Multinomial = 0.08) and current mood disorder (χ^2^ [3, N = 200] = 5.48, *p* = 0.14, BF_10_ Independent Multinomial = 0.21). 

### 3.3. AMPD Profiles

**Figure 2:**
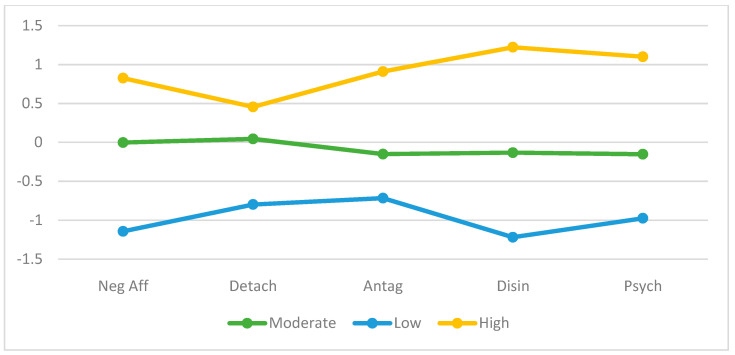
Profile plot for Pathological Personality Traits with AMPD-Only Profiles. *Note.* Standardized mean scores (M = 0, SD = 1) of the Alternative Model of Personality across three profiles.

Like the extraction with FFM-only profiles, the optimal number of profiles was selected based on theoretical considerations, profile interpretability, and the comparison of alternative profile solutions (see Table 4). The three-profile solution was the most suitable given theoretical considerations and it demonstrated the highest classification accuracy as the BIC reaches a minimum and entropy a maximum at three profiles (see Figure 2). The High Psychopathology group (22.1% of respondents; 39.5% male, 60.46% female) was between the ages of 20 to 56 (M = 30.73; SD = 8.89) and consisted of the highest scores across all pathological personality domains compared to the other two profiles. The Moderate Psychopathology group (62.3% of respondents; 54.5% male, 45.5% female) was between the ages of 18 to 87 (M = 41.23; SD = 13.71) and revealed moderate scores across all personality domains compared to the other two profiles. The Low Psychopathology group (n = 30, 43.33% male, 56.67% female) was between the ages of 19 and 71 (M = 45.43; SD = 13.52) and showed the lowest scores across all personality domains compared to the other two profiles. The most critical variable was disinhibition as evinced by the largest effect size estimates VS-MPR (ηp^2^ = 0.60; VS-MPR = 3.46 × 1035). The least essential variables were detachment (ηp^2^ = 0.24; VS-MPR = 2.02 × 10 + 9) and antagonism (ηp^2^ = 0.24; VS-MPR = 2.85 × 10 + 9), with the smallest effect size and VS-MPR.

Profiles did not differ based on gender (χ^2^[3, N = 194] = 5.92, *p* = 0.21, BF_10_ Independent Multinomial = 0.28). Age predicted profile membership (P[M] = 0.50, P[M|data] = 1.00, BF_M_ = 12,364, error % = 0.02). With posterior odds corrected for multiple testing and prior odds set as 0.59 (Westfall et al. 1997), there was extreme evidence that the High Psychopathology group were younger than the Moderate Psychopathology group (Posterior Odds = 1910; BF_10_ = 3251, error% < 0.001) and Low Psychopathology group (Posterior Odds = 24,011, BF_10_ = 40,877, errors% < 0.001). No evidence was found for other age differences between Moderate and Low Psychopathology profiles.

### 3.4. Associations of AMPD-Only Profiles with Clinical Symptoms and Cognitive Ability Measures

After explaining the error variance attributable to gender and age, profile membership was associated with enhanced problematic gambling. As such, the model with profile and gender (P[M] = 0.13, P[M|Data] = 0.53, BF_M_ = 7.89, BF_10_ = 1359, error% = 1.78) outperformed other combinations of the profile, gender, and age. Post hoc tests indicated that High Psychopathology (M = 3.32, SD = 5.29) scored higher than Moderate Psychopathology (M = 1.05, SD = 2.20; Posterior Odds = 42.07, BF_10,U_ = 71.63, error% < 0.001) and Low Psychopathology on the PGSI (M = 1.55, SD = 4.41; Posterior Odds = 12.19, BF_10,U_ = 20.75, error% < 0.001). There was a significant relationship between profile membership and problematic gambling with medium to large effect sizes, χ^2^(3, N = 199) = 13.92, *p* < 0.001, VS-MPR = 55.62, Cramer’s V = 0.26, BF_10_ = 25.13. The High Psychopathology group (z = 3.71) were likelier than expected to meet the cut-off for problematic gambling. 

When accounting for the error variance and the effects of gender and age, profile membership was associated with problematic alcohol use. Specifically, the model with profile (P[M] = 0.13, P[M|Data] = 0.49, BF_M_ = 6.63, BF_10_ = 9.69, error% = 0.01) was more probable than other combinations of gender, age, and profile. Post hoc comparisons suggested that the High Psychopathology profile (M = 10.77, SD = 8.27) scored higher than Moderate (M = 7.17, SD = 8.57; Posterior Odds = 14.05, BF_10,U_ = 23.91, error% < 0.001) and Low Psychopathology profiles (M = 4.55, SD = 5.51; Posterior Odds = 221, BF_10,U_ = 376, error% < 0.001) on the AUDIT. When using cut-offs (AUDIT ≥ 5), a Chi-Square Test of Independence showed a significant link between profile membership and alcohol use, χ^2^(3, N = 199) = 10.89, *p* < 0.01, VS-MPR = 15.65, Cramer’s V = 0.23, BF_10_ = 11.87. The High Psychopathology group (z = 3.26) was more likely to meet the cut-off for problematic alcohol use than expected.

AMPD-only profile membership did not predict matrix reasoning or verbal abilities. For matrix reasoning, the null model (P[M] = 0.50, P[M|Data] = 0.91, BF_M_ = 9.83, BF_10_ = 0.10) outperforms the profiles model (P[M] = 0.50, P[M|Data] = 0.09, BF_M_ = 0.10, BF_10_ = 0.10, error% = 0.02). Similarly, the null model (P[M] = 0.50, P[M|Data] = 0.92, BF_M_ = 11.92, BF_10_ = 1.00) for verbal reasoning outperforms the profiles model (P[M] = 0.50, P[M|Data] = 0.08, BF_M_ = 0.08, BF_10_ = 0.08, error% = 0.02). 

There was no significant relationship between profile membership and lifetime anxiety disorder diagnosis, χ^2^(2, N = 200) = 7.15, *p* = 0.38, BF_10_ = 0.13. There was a significant association between AMPD-only profile membership and current anxiety disorder diagnosis, χ^2^(2, N = 200) = 13.00, *p* < 0.01, VS-MPR = 37.57, Cramer’s V = 0.25, BF_10_ = 28.89. As expected, the High Psychopathology group was more likely than expected to meet criteria for an anxiety disorder (z = 3.04), whereas the Low Psychopathology group was less likely than expected to meet criteria for an anxiety disorder (z = −2.58). There was a significant association between profile membership and lifetime substance use disorder, χ^2^(2, N = 201) = 8.38, *p* < 0.05, VS-MPR = 5.79, Cramer’s V = 0.20, BF_10_ = 2.32, and current substance use disorder, χ^2^(3, N = 199) = 8.17, *p* < 0.05, VS-MPR = 5.35 Cramer’s V = 0.20, BF_10_ = 4.63. In both cases, the High Psychopathology group was more likely to meet the criteria, whereas the Low Psychopathology group were less likely to meet the criteria for substance use disorder (Table 3). There was no evidence that AMPD profile membership was associated with lifetime mood disorder, χ^2^(2, N = 200) = 1.91, *p* = 0.38, BF_10_ = 0.11, or current mood disorder, χ^2^(2, N = 200) = 4.28, *p* = 0.12, BF_10_ = 0.36. 

### 3.5. FFM–AMPD Profiles

The five factors from the BFAS and PID-5 were finally entered in a latent profile analysis (Table 5). The five-profile solution had lowest BIC of all profiles and the highest entropy. However, examination of the profiles produced by the five-profile solutions suggested the “adaptive profile” from the four-profile solution was split into two separate profiles based on severity (i.e., one endorsing more psychopathology, one endorsing less). Thus, the more parsimonious four-profile solution was the most suitable given that it created distinct, practically meaningful profiles, yet showed a better fit for the data than the three-profile solution (Spurk et al. 2020). 

FFM-only and AMPD-only profiles extracted earlier in our samples were named according to profiles extracted in previous investigations. The profiles extracted from the combination of FFM and AMPD traits required a different naming system given that these present profiles have not been previously extracted (i.e., previous studies used a different and narrower set of traits from which profiles were extracted). The profiles were labelled adhering to naming practices specific to a theoretical framework (Weller et al. 2020). The Hierarchical Taxonomy of Psychopathology (HiTOP) consortium outlines significant psychopathology dimensions at the higher-order spectra: internalizing, somatoform, disinhibited externalizing, antagonistic externalizing, thought disorder, and detachment. The spectra can be combined into larger superspectra: emotional dysfunction (internalizing and somatoform), externalizing (disinhibited and antagonistic), and psychosis (thought disorder and detachment; Kotov et al. 2017, 2020; Markon et al. 2011). 

**Figure 3:**
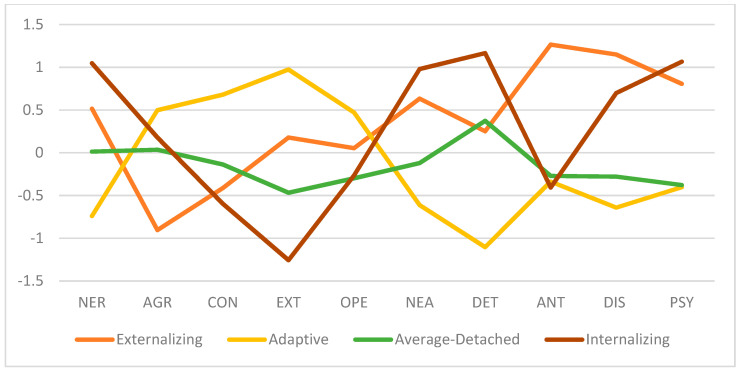
Profile plot for Five Factors for FFM–AMPD Combined Profiles. *Note.* Standardized mean scores (M = 0, SD = 1) of the Five Factor Model of Normative Personality and Alternative Model of Personality across four profiles.

Figure 3 shows a visual depiction of the means across profiles. The Externalizing profile members (21.5% of respondents; 46.3% male, 53.7% female) were between the ages of 20 to 56 (M = 32.31; SD = 10.37) and labeled accordingly based on the lowest scores in agreeableness and highest scores in antagonism and disinhibition. The Adaptive profile (28.2% of respondents, 45.5% male, 54.5% female) between the ages of 19 and 71 (M = 39.67; SD = 12.86) has the lowest neuroticism, negative affectivity, detachment, antagonism, disinhibition, and psychoticism while exhibiting the highest agreeableness, conscientiousness, extraversion, and openness to experience compared to its counterparts. The Average-Detached profile (40% of respondents, 57.7% male, 42.3% female) was between the ages of 18 to 73 (M = 43.27; SD = 13.99) and shows moderate scores across all domains with the lowest scores in openness to experience (tied with Internalizing-Thought disorder profile) and psychoticism (tied with the Adaptive profile). The Average-Detached profile was labelled as such given its maxima is in the detachment domain. The Internalizing-Thought disorder profile (10.3% of respondents, 35% male, 65% female) between the ages of 18 to 73 (M = 43.27; SD = 13.99) had the lowest extraversion and openness to experience and highest neuroticism, negative affectivity, detachment, and psychoticism compared to other profiles. In this latent profile analysis, the most important variable is detachment as evinced with the largest effect size and VS-MPR (ηp^2^ = 0.61; VS-MPR = 4.55 × 10 + 34), reflecting larger differences between profile memberships. The least important variable is openness-to-experience, with the smallest effect size and VS-MPR (ηp^2^ = 0.09; VS-MPR = 88.35). Appendix A shows details of these analyses. 

Similar to previous findings, profiles did not differ based on gender (χ^2^[3, N = 194] = 4.30, *p* = 0.23, BF_10_ Independent Multinomial = 0.11). Age predicted profile membership (P[M] = 0.50, P[M|data] = 0.99, BF_M_ = 77.31, error % < 0.01). With posterior odds corrected for multiple testing and prior odds set as 0.41 (Westfall et al. 1997), there was extreme evidence that the Externalizing profile was younger than the Average-Detached profile (Posterior Odds = 395; BF_10_ = 955, error% < 0.001) and strong evidence that the Externalizing group was younger than the Adaptive profile (Posterior Odds = 4.66, BF_10_ = 11.26, errors% < 0.001). No evidence was found for other age differences for other profiles.

### 3.6. Associations of FFM–AMPD Profiles with Clinical Symptoms

When accounting for the error variance and the effects of gender and age, profile membership was associated with problematic gambling (Table 6). The model with profile, gender, and age (P[M] = 0.13, P[M|Data] = 0.51, BF_M_ = 7.25, BF_10_ = 389.21, error% = 1.33) was compared to the gender and age model (P[M] = 0.13, P[M|data] = 0.04, BF_M_ = 0.32, BF_10_ = 33.00, error% < 0.01). The model with profile group was preferred in predicting problematic gambling to the covariates only model by a factor of 12 (389/33 ≃ 11.79). Post hoc tests show that the Externalizing profile (M = 3.19; SD = 5.23) scored higher than Average-Detached (M = 0.96, SD = 2.20; Posterior Odds = 30.37, BF_10, U_ = 73.33, error% < 0.001) and Adaptive (M = 1.26, SD = 3.31; Posterior Odds = 13.02, BF_10, U_ = 31.43, error% < 0.001) profiles. When using cut-offs for problematic gambling (PGSI ≥3), a Chi-Square Test of Independence revealed a significant relationship between the profile memberships and problematic gambling with medium effect sizes, χ^2^(3, N = 199) = 12.21, *p* < 0.01, VS-MPR = 10.96, Cramer’s V = 0.25, BF_10_ = 1.20. The Externalizing profile members (z = 3.47) were more likely to meet the cut-off for problematic gambling compared to the other groups. 

There was only anecdotal evidence that profile membership was linked with problematic alcohol use (P[M] = 0.13, P[M|Data] = 0.28, BF_M_ = 2.74, BF_10_ = 2.57, error% < 0.001). However, there was a significant relationship between problematic alcohol use and profile membership with medium effect sizes when using categorical cut-offs (AUDIT ≥ 5), χ^2^(3, N = 199) = 11.80, *p* < 0.01, VS-MPR = 9.41, Cramer’s V = 0.24, BF_10_ = 4.91. The Externalizing profile was more likely (z = 2.64) to meet the criteria than expected. 

Based on Bayesian ANOVA, there was no evidence that the FFM–AMPD profile membership was associated with matrix reasoning and verbal abilities. For verbal comprehension, there was no evidence that the profiles model (P[M] = 0.50, P[M|Data] = 0.04, BF_M_ = 0.04, BF_10_ = 0.04, error% < 0.01) outperforms the null model (P[M] = 0.50, P[M|Data] = 0.96, BF_M_ = 25.52, BF_10_ = 1.00). Similarly, there was no evidence that the profiles model (P[M] = 0.50, P[M|Data] = 0.04, BF_M_ = 0.04, BF_10_ = 0.04, error% < 0.01) outperforms the null model (P[M] = 0.50, P[M|Data] = 0.96, BF_M_ = 25.24, BF_10_ = 1.00) for matrix reasoning.

There were no significant associations between profile membership and lifetime DSM diagnosis, including lifetime anxiety disorder (χ^2^[3, N = 200] = 3.97, *p* = 0.26, BF_10_ Independent Multinomial = 0.09), lifetime substance use disorder (χ^2^[3, N = 201] = 6.92, *p* = 0.07, BF_10_ Independent Multinomial = 0.37), and lifetime mood disorder (χ^2^[3, N = 200] = 3.96, *p* = 0.27, BF_10_ Independent Multinomial = 0.12).

Profile membership was associated with current anxiety disorder diagnosis (χ^2^[3, N = 200] = 18.58, *p* < 0.001, Cramer’s V = 0.30, VS-MPR > 100, BF_10_ Independent Multinomial = 236.93). The Internalizing-Thought disorder profile was more likely than expected to meet the criteria for an anxiety disorder (z = 2.83), whereas the Adaptive group were less likely to meet the criteria for a current anxiety disorder (z = 3.77). Profile membership was also significantly associated with current substance use disorder (χ^2^[3, N = 199] = 8.29, *p* < 0.05 Cramer’s V = 0.20, VS-MPR = 2.84, BF_10_ Independent Multinomial = 1.10). The Externalizing profile was more likely than expected to meet the criteria for a current substance use disorder (z = 2.64). Profile membership was associated with current mood disorder (χ^2^ [3, N = 200] = 8.57, *p* < 0.05, Cramer’s V = 0.21, VS-MPR = 3.09, BF_10_ Independent Multinomial = 0.98). The Internalizing group were more likely (z = 2.21) and the Adaptive group were less likely (z = −2.27) to meet the criteria for a current mood disorder.

### 3.7. Comparisons of Profiles

Significant overlaps existed between the FFM-only and the FFM–AMPD combined profiles, χ^2^ [9, N = 201] = 160.58, *p* < 0.001, Cramer’s V = 0.52, VS-MPR > 100, BF_10_ Independent Multinomial > 100 (Table 6). Specifically, the FFM-only Undercontroller profile overlaps largely with the FFM–AMPD Externalizing profile, whereas the FFM-only Resilient profile corresponds with the FFM–AMPD combined Adaptive profile. The FFM-only Overcontrollers profile resembles both the FFM–AMPD combined Average-Withdrawn and Internalizing-Thought disorder profiles. The FFM-only Ordinary profile is more widely dispersed compared to the other profiles but shows the most overlap (non-significant) with the Adaptive profile. 

When comparing AMPD-only and the FFM–AMPD combined profiles, there were significant connections between the profiles χ^2^ [6, N = 201] = 172.32, *p* < 0.001, Cramer’s V = 0.65, VS-MPR > 100, BF_10_ Independent Multinomial > 100). Specifically, the FFM–AMPD Externalizing and Internalizing profiles overlap largely with the AMPD-only High Psychopathology group. The FFM–AMPD Adaptive profile corresponds with the AMPD-only Low Psychopathology group whereas the FFM–AMPD Average-Withdrawn profile intersects with the AMPD-only Moderate Psychopathology group. 

Finally, there were significant associations between the FFM-only and AMPD-only profiles, χ^2^ [6, N = 201] = 86.43, *p* < 0.001, Cramer’s V = 0.46 VS-MPR > 100, BF_10_ Independent Multinomial > 100. The FFM-only Undercontroller profile largely overlaps with the AMPD-only High Psychopathology group whereas the FFM-only Resilient profile overlaps with Low Psychopathology. The FFM-only Overcontroller and Ordinary profiles demonstrate significant overlap with the AMPD-only Moderate Psychopathology group.

## 4. Discussion

Our main goal for this investigation was to attempt to extract latent profiles from FFM and AMPD both together and separately to determine whether the additional construct coverage of AMPD personality traits may add additional information to the FFM. In the past decade, the AMPD has gained considerable attention as it provides information on personality psychopathology with practical and significant clinical implications (Clark and Watson 2022). Using best practices with latent profile analyses, four profiles (i.e., Undercontroller, Overcontroller, Ordinary, and Resilient) were extracted when only the FFM was used, and AMPD-only profiles uncovered three profiles of High, Moderate, and Low Psychopathology. When considering both the FFM and AMPD, four profiles were revealed as Internalizing-Thought disorder, Externalizing, Average-Detached, and Adaptive profiles. Notably, entropy, as a measure of how accurately the model defines profiles (Celeux and Soromenho 1996; Wang et al. 2017), was the highest in the FFM–AMPD combined profiles (entropy = 0.84) compared to FFM-only (entropy = 0.66) and AMPD-only (entropy = 0.79). The minimal value of the diagonal of the average latent class probabilities for the most probable profile was also highest in FFM–AMPD profiles. These results suggest a better fit of the model with the data when using FFM–AMPD profiles. Given that entropy indicates the mixture model’s ability to return well-separated profiles, the FFM-only profiles may not provide distinguished separation between profiles. Hence, future studies should investigate whether FFM traits may be combined with other indicators for profile formation to enhance entropy. 

Our first research objective was to evaluate whether a four-profile solution of the FFM would emerge in a clinical sample as it did previously in community and clinical samples. The characteristics of the four profiles extracted from the FFM-only profiles were broadly consistent with the average level and shape compared to other community and university samples with Yin et al.’s (2021) systematic review. The Undercontroller profile members demonstrated low agreeableness and conscientiousness, whereas the Overcontrollers group exhibited high neuroticism and low extraversion and openness-to-experience. The Resilient group emerged with low neuroticism and high agreeableness, openness to experience, extraversion, and conscientiousness. Consistent with other latent profiles extracted with the FFM, an Ordinary group also emerged with mid-ranged symptoms across all domains compared to other profiles (Kinnunen et al. 2012; Zhang et al. 2015; Min and Su 2020). Given the dimensional nature of these constructs, individuals classified under these symptom profiles with mid-ranged symptoms may not be experiencing significant personality psychopathology but are also not free of symptoms. 

Latent profile analyses with only AMPD traits revealed three AMPD profiles with identical shapes of varying levels of psychopathology (i.e., high, moderate, and low). These findings reflect other latent profiles that found similar high and low personality psychopathology with similar shapes (e.g., Ahmed et al. 2021; Li et al. 2019; Tabak and Weisman de Mamani 2013). Like the p factor in psychopathology (Caspi et al. 2014; Caspi and Moffitt 2018), the p factor in personality disorders as an integrated personality trait may explain why all five pathological personality traits are highly comorbid (Asadi et al. 2021). Of note, these results differ from that of Hanegraaf et al. (2022), who extracted four profiles using the AMPD. Notably, Hanegraaf et al. (2022) utilized the short version of the PID-5 and recruited an MTURK sample as opposed to a treatment-seeking clinical sample in the present study. In the present study, four profiles were not extracted given the increase in BIC and a large decrease in entropy. Based on sample characteristics (i.e., age, gender, clinical status), the number and type of profiles may differ. Future studies should replicate findings to reveal whether community or clinical samples may differ based on the number of profiles extracted.

The second research objective was to extract profiles from both FFM and AMPD and compare them to FFM-only and AMPD-only profiles. This is the first study to assess personality profiles that both the FFM and AMPD provided. Results corroborate that the sample size and characteristics of the FFM–AMPD profiles were different from FFM-only and AMPD-only. Within the FFM-only profiles, the present study found that agreeableness and openness to experience were the most and least important variables, respectively, when distinguishing between profiles. Likewise, Yin et al. (2021) found across 34 studies that openness to experience is the least important for the profile classification but found neuroticism as the most important dimension. For AMPD, the most important variable is disinhibition while the least important variables were detachment and antagonism. In contrast, the most important variable is detachment while the least important is openness to experience in the FFM–AMPD profiles. Remarkably, the detachment dimension showed high scores in the Externalizing and Internalizing-Thought disorder profiles, moderate scores with the Average-Detached profile, and low scores in the Adaptive profile. For AMPD, detachment was high, moderate, and low in the High Psychopathology, Moderate Psychopathology, and Low Psychopathology group, respectively. The distribution of trait scores differs more substantively across profiles in the FFM–AMPD combined profiles. These findings speak to the differential results in latent profiles when FFM and AMPD measures are combined, despite the substantive conceptual overlap in four of five traits between the FFM and AMPD. Importantly, 19 of 42 (45.2%) individuals classified as Externalizing were Undercontrollers while 19 of 28 (67.86%) individuals classified as Undercontrollers were part of the Externalizing group. Moreover, 18 of 20 (90%) Internalizing-Thought disorder members were classified as Overcontrollers, yet 18 of 79 Overcontrollers (22.78%) were classified as Internalizing-Thought disorder. In other words, almost all members of the Internalizing group are Overcontrollers, but only a quarter of Overcontrollers have Internalizing-Thought disorder membership. Combined with the findings of higher neuroticism scores in the Internalizing-Thought disorder group and that this group was associated with current mood disorder diagnosis, the Internalizing-Thought disorder profile may be extracted as a group with more severe psychopathology within the Overcontrollers. 

No gender differences were found across all profiles, which is contrary to expectations that males and females are overrepresented among Undercontrollers and Overcontrollers, respectively (e.g., Akse et al. 2004; Asendorpf et al. 2001; Dubas et al. 2002). Moreover, verbal and matrix reasoning scores did not differ even though lower IQ in childhood and adulthood were associated with enhanced risk of psychopathology (Koenen et al. 2009; Melby et al. 2020). Executive function and cognitive reserve are implicated across psychiatric disorders and may, therefore, not discriminate well at this level of resolution. Specifically, this study recruited a treatment-seeking clinical sample in a mental health hospital, suggesting that almost all patients are expected to be experiencing significant internalizing or externalizing psychopathology. Perhaps the addition of a community-based sample who completed the questionnaires and cognitive tasks into the analyses may indicate substantive differences across cognitive ability between groups. Future studies should uncover whether these profiles may be associated with differential scores with the full WAIS battery in a mixed community and clinical sample (Wechsler 1997). 

Our third goal was to examine to whether profiles showed differential patterns of association with a set of external criterion variables. In the FFM-only profiles, Undercontrollers were more likely to report problematic gambling and Overcontrollers were more likely to be diagnosed with an anxiety disorder. Results were consistent with findings that classification as an undercontroller at age 3 predicted future problematic gambling habits in adulthood, even when controlling for socio-economic status and childhood IQ (Slutske et al. 2012). For AMPD-only profiles, the High Psychopathology profile was more likely to engage in problematic gambling and alcohol use than other groups. Members of the High Psychopathology group were also more likely diagnosed with a current anxiety disorder and substance use disorder, but not a mood disorder. For FFM–AMPD, the Internalizing-Thought disorder profile was more likely to meet the criteria for a current mood and anxiety disorder. The Externalizing profile was more likely to meet the criteria for a current substance use disorder and engage in problem gambling and alcohol use. 

In contrast to FFM-only profiles that were not associated with current substance use disorder and mood disorder, the FFM–AMPD combined profiles may have better convergent and discriminant validity for DSM-relevant psychopathology. Notably, the profiles were only associated with current, but not lifetime, diagnosis, suggesting that these profiles reflected current symptoms, difficulties, and impairments experienced. Profiles may be associated with current diagnoses, but not lifetime diagnoses, because the PID-5 assesses significant personality dysfunction at present and personality may change over the course of the lifetime and throughout treatment (Roberts et al. 2006, 2017). Lifetime diagnoses may be indicative of acute symptom changes at a specific time, which may not be reflective of the individual‘s current disposition. The combined profiles may be meaningful and clinically useful for the clinical population under investigation.

### Limitations and Future Directions

The present study is not without some limitations. First, the study used self-report measures as indicators for latent profiles, which may be predisposed to social desirability or degree of insight for the individual. Future studies should evaluate whether informant reports of the five-factor model of personality and alternative model of personality disorder traits produce similar profiles found in this study (Markon et al. 2013). Second, the cross-sectional nature of this study limits any causal extrapolations regarding the influences of these profile characteristics on clinical symptoms and mental health disorder diagnoses. Of note, the individuals falling in the High Psychopathology, Undercontrollers, and Externalizing profiles were younger than others, which was consistent with previous findings (Specht et al. 2014). Undercontrollers tend to experience increased agreeableness over time, which may suggest improvements with poor behavioural control with age (Klimstra et al. 2010; Morizot and Le Blanc 2005). Furthermore, externalizing psychopathology in youth is linked with developing mood disorders in adulthood (Loth et al. 2014). Future studies may assess whether heterotypic continuity occurs in personality profiles, such as that of psychiatric disorders across the lifespan (Lavigne et al. 2014; Lahey et al. 2014). Of note, the present study excluded patients with current or lifetime history of psychotic disorder. Samples with patients who have or are currently experiencing psychosis may have a broader range of psychoticism in AMPD-based profile formation. Third, the associated measures evaluated in the present study were limited to cognitive ability, problematic gambling, problematic alcohol use, and DSM diagnoses. Future studies should explore whether these profiles may be able to distinguish other behavioural addictions, such as problematic gaming (e.g., Richard et al. 2020), which are associated with both internalizing and externalizing symptoms (Lau et al. 2018). Future studies should also investigate whether self-reported profiles align with specific neurodevelopmental processes and mechanisms (Casey et al. 2014). Fourth, the stability of the latent profile solutions needs to be replicated, as previous findings revealed unstable or unreliable latent class solutions across individuals (Freudenstein et al. 2019). Future studies should replicate these findings and utilize both raw and age-corrected scores for intelligence measures. Lastly, the present study utilized the classification scheme of personality disorder consistent with the PID-5 aligned with DSM-5 classification. Using the International Classification of Disease (11th ed.; ICD-11) personality disorder (PD) conceptualization of psychopathology, Sellbom et al. (2020) proposed five trait domains that may be measured with the PID-5: Negative Affectivity, Detachment, Dissociality, Disinhibition, and Anankastia. Future findings should consider whether similar or conceptually distinct personality profiles emerge using these different classifications of pathological personality traits.

## 5. Conclusions

In conclusion, the present study extracted profiles based on the FFM, AMPD, and FFM–AMPD combined traits, tested the criterion validity of these extracted profiles, and compared how these profiles may overlap with one another. Findings revealed evidence that the FFM–AMPD combined trait domain profiles revealed multiple unique subgroups in a clinical sample, which were uniquely linked with DSM diagnoses, cognitive ability, and clinical symptoms. Future research should investigate whether dimensional profile assessment with both the FFM and AMPD informs better evaluation of personality strengths and impairment. These results will enable researchers and clinicians to identify symptomatology profiles based on dimensional assessment, and to evaluate their clinical utility.

## Figures and Tables

**Table 1:** Means, Standard Deviations, Reliabilities, and Bivariate Bayesian Pearson’s rho Correlations between the Five-Factor Model and Alternative Model of Personality Disorder Traits.

	Mean	SD	N	A	C	E	O	Neg	Det	Ant	Dis	Psy
N	3.58	0.74	0.91 [0.89, 0.93]									
A	4.08	0.56	−0.10	0.85 [0.81, 0.87]								
C	3.10	0.64	−0.28 ***	0.19	0.85 [0.82 0.88]							
E	3.15	0.76	−0.31 ***	0.14	0.29 ***	0.90 [0.89, 0.92]						
O	3.86	0.59	−0.07	0.17	−0.02	0.41 ***	0.84 [0.80, 0.87]					
Neg	1.64	0.61	0.76 ***	−0.07	−0.29 ***	−0.20	−0.04	0.88 [0.85, 0.90]				
Det	1.35	0.60	0.42 ***	−0.23 *	−0.28 ***	−0.70 ***	−0.23 *	0.31 ***	0.86 [0.83, 0.88]			
Ant	0.73	0.54	0.19	−0.65 ***	−0.16	0.14	0.04	0.27 ***	0.09	0.87 [0.85, 0.89]		
Dis	1.24	0.55	0.44 ***	−0.39 ***	−0.54 ***	−0.10	−0.04	0.49 ***	0.24 **	0.52 ***	0.81 [0.77, 0.84]	
Psy	0.93	0.59	0.36 ***	−0.18	−0.26 ***	−0.08	0.15	0.44 ***	0.29 ***	0.37 ***	0.57 ***	0.87 [0.84, 0.89]

Note. * BF_10_ > 10, ** BF_10_ > 30, *** BF_10_ > 100 for Bayesian Pearson’s rho correlations. Bayesian Scale Reliability McDonald’s Omega posterior means are listed in the diagonal with 95% Credible Interval (CI) lower bound and upper bound. N = Neuroticism, A = Agreeableness, C = Conscientiousness, E = Extraversion, O = Openness to Experience. Neg = Negative Affectivity. Det = Detachment, Ant = Antagonism, Dis = Disinhibition, Psy = Psychoticism.

**Table 2:** Model fit indices, class size, and class probabilities for FFM-only latent profile membership.

Profiles	AIC	BIC	SABIC	BLRT (p)	Entropy	N Min	N Max	Prob Min	Prob Max
2-Profile	2801	2853	2803	78.52 (0.01)	0.65	0.36	0.64	0.85	0.92
3-Profile	2794	2866	2797	18.93 (0.01)	0.64	0.22	0.52	0.77	0.90
4-Profile	2777	2870	2781	28.26 (0.01)	0.66	0.14	0.39	0.76	0.86
5-Profile	2768	2880	2772	21.59 (0.02)	0.70	0.05	0.36	0.66	0.88

Note. AIC = Akaike Information Criterion; BIC = Bayesian Information Criterion; SABIC = Sample Size Adjusted Bayesian Information Criterion. N Min: Proportion of participants designated to the smallest profile (most probable profile membership). N Max: Proportion of participants designated to the largest profile (most probable profile membership). Prob Min: Minimal of the diagonal of the average latent class probabilities for most probable profile. Prob. Max: Maximum of the diagonal of the average latent class probabilities for most probable profile.

**Table 3:** Cross-Tabulations of Profile Membership and Criteria met for Current DSM Diagnoses.

	ANXIETY DISORDER NOT MET	ANXIETY DISORDER MET	SUBSTANCE USE DISORDER NOT MET	SUBSTANCE USE DISORDER MET	MOOD DISORDER NOT MET	MOOD DISORDER MET
FFM ONLY PROFILES						
UNDERCONTROLLERS	19 (0.28)	8 (−0.28)	20 (−1.38)	8 (1.38)	21 (1.28)	6 (−1.28)
RESILIENT	31 (2.28) *	6 (−2.28)	33 (1.82)	3 (−1.82)	28 (1.24)	9 (−1.24)
OVERCONTROLLERS	46 (−2.39)	33 (2.39) *	66 (0.77)	13 (−0.77)	46 (−2.13)	33 (2.13) *
ORDINARY	40 (0.42)	17 (−0.42)	42 (−1.33)	14 (1.33)	39 (0.27)	18 (−0.27)
AMPD ONLY PROFILES						
HIGH PSYCHOPATHOLOGY	21 (−3.04)	22 (3.04) *	31 (−2.00)	11 (2.00) *	24 (−1.76)	19 (1.76)
MODERATE PSYCHOPATHOLOGY	87 (0.63)	38 (−0.63)	100 (−0.12)	24 (0.12)	85 (0.39)	40 (−0.39)
LOW PSYCHOPATHOLOGY	28 (2.58) *	4 (−2.58)	30 (2.45) *	1.00 (−2.45)	25 (1.46)	7 (−1.46)
FFM–AMPD PROFILES						
EXTERNALIZING	26 (−0.71)	15 (0.71)	28 (−2.64)	14 (2.64) *	28 (0.20)	13 (−0.20)
ADAPTIVE	50 (3.77) *	7 (−3.77)	50 (1.88)	6 (−1.88)	45 (2.27) *	12 (−2.27)
AVERAGE-WITHDRAWN	52 (−1.16)	30 (1.16)	66 (0.17)	15 (−0.17)	52 (−0.90)	30 (0.90)
INTERNALIZING	8 (−2.83)	12 (2.83) *	17 (0.49)	3 (−0.49)	9 (−2.21)	11 (2.21) *

*Note.* Number represents number of participants meeting or not meeting criteria for DSM diagnoses. Number in brackets represents the standardized residual. The standardized residuals are computed by (observed − expected)/sqrt(expected × [1 − row marginal proportion] × [1 − column marginal proportion]). * represents >1.96 in standard residuals.

**Table 4:** Model Fit Indices, Class Size, and Class Probabilities for AMPD-Only Latent Profile Membership.

Profiles	AIC	BIC	SABIC	BLRT (p)	Entropy	N Min	N Max	Prob Min	Prob Max
2-Profile	2716	2768	2718	163.50 (.01)	.73	.34	.66	.87	.94
3-Profile	2670	2743	2673	57.71 (.01)	.79	.16	.62	.83	.94
4-Profile	2662	2754	2665	20.24 (.01)	.72	.14	.51	.60	.89
5-Profile	2643	2755	2647	30.83 (.01)	.75	.09	.41	.79	.90

*Note.* AIC = Akaike Information Criterion; BIC = Bayesian Information Criterion; SABIC = Sample Size Adjusted Bayesian Information Criterion. N Min: Proportion of participants designated to the smallest profile (most probable profile membership). N Max: Proportion of participants designated to the largest profile (most probable profile membership). Prob Min: Minimal of the diagonal of the average latent class probabilities for most probable profile. Prob. Max: Maximum of the diagonal of the average latent class probabilities for most probable profile.

**Table 5:** Model fit indices, class size, and class probabilities for FFM–AMPD Combined latent profile membership.

Profiles	AIC	BIC	SABIC	BLRT (p)	Entropy	N Min	N Max	Prob Min	Prob Max
2-Profile	5475	5577	5479	281.43 (.01)	.81	.33	.67	.92	.96
3-Profile	5349	5487	5354	148.01 (.01)	.82	.24	.43	.90	.95
4-Profile	5305	5480	5313	65.26 (.01)	.84	.10	.41	.87	.92
5-Profile	5211	5412	5219	116.57 (.01)	.86	.10	.49	.86	.95
6-Profile	5191	5438	5201	42.27 (.01)	.85	.10	.33	.82	.95

*Note*. AIC = Akaike Information Criterion; BIC = Bayesian Information Criterion; SABIC = Sample Size Adjusted Bayesian Information Criterion. N Min: Proportion of participants designated to the smallest profile (most probable profile membership). N Max: Proportion of participants designated to the largest profile (most probable profile membership). Prob Min: Minimal of the diagonal of the average latent class probabilities for most probable profile. Prob. Max: Maximum of the diagonal of the average latent class probabilities for most probable profile.

**Table 6:** Cross-Tabulation of FFM-Only and FFM–AMPD Combined Profiles, AMPD-Only and Combined Profiles, and FFM-Only and Combined.

AMPD Only and FFM Only	Under-Controllers	Resilients	Over-Controllers	Ordinary	Total	Externalizing	Adaptive	Average-Withdrawn	Internalizing	Total
High Psychopathology	14 (3.88) *	0 (−3.56)	19 (.60)	11 (−.56)	44	33 (9.99) *	0 (−4.72)	0 (−6.23)	11 (3.77) *	44
Moderate Psychopathology	13 (−1.85)	14 (−3.38)	56 (2.05) *	42 (2.11) *	125	9 (−6.12)	31 (−1.44)	76 (7.40) *	9 (−1.67)	125
Low Psychopathology	1 (−1.93)	23 (8.51) *	4 (−3.39)	4 (−2.17)	32	0 (−3.17)	26 (7.24) *	6 (−2.77)	0 (−2.05)	32
Total	28	37	79	57	201	42	57	82	20	201
FFM only and Combined	Under-controllers	Resilients	Over-controllers	Ordinary	Total	-	-	-	-	-
Externalizing	19 (6.59) *	0 (−3.46)	12 (−1.60)	11 (−.35)	42	-	-	-	-	-
Adaptive	3 (−2.23)	34 (9.49) *	0 (−7.18)	20 (1.33)	57	-	-	-	-	-
Average-Detached	6 (−2.25)	3 (−4.48)	49 (4.93) *	24 (.24)	82	-	-	-	-	-
Internalizing	0 (−1.90)	0 (−2.24)	18 (4.89) *	2 (−1.92)	20	-	-	-	-	-
Total	28	37	79	57	201	-	-	-	-	-

*Note.* Number represents actual count. Number in brackets represents the standardized residual. The standardized residuals are computed with the following equation: (observed − expected)/sqrt(expected × [1 − row marginal proportion] × [1 − column marginal proportion]). * represents >1.96 as a z-score for standardized residuals.

## Data Availability

The data presented in this study are available on request from the last author, Lena C. Quilty. The data are not publicly available.

## Appendix A

**Table A1:** Means, Standard Deviations, ANCOVA F statistic, Vovk-Sellke Maximum p-Ratio, and eta squared of FFM-Only Profiles.

	Mean (SD) of Undercontoller Profile	Mean (SD) of Resilient Profile	Mean (SD) of Overcontroller Profile	Mean (SD) of Ordinary Profile	F Statistic	VS-MPR	η^2^	η_p_^2^
Openness	3.81 (0.53)	3.95 (0.54)	3.48 (0.49)	4.35 (0.42)	*F* (3, 187) = 30.05, *p* < 0.001	1.48 × 10^+13^	0.32	0.33
Neuroticism	3.62 (0.91)	2.82 (0.60)	3.86 (0.56)	3.75 (0.57)	*F* (3, 187) = 28.69, *p* < 0.001	4.16 × 10^+12^	0.28	0.32
Agreeableness	3.10 (0.31)	4.50 (0.34)	4.06 (0.37)	4.29 (0.36)	*F* (3, 187) = 86.6, *p* < 0.001	1.31 × 10^+32^	0.56	0.58
Conscientiousness	2.92 (0.69)	3.93 (0.30)	2.84 (0.55)	2.99 (0.43)	*F* (3, 187) = 38.79, *p* < 0.001	5.53 × 10^+16^	0.38	0.38
Extraversion	3.26 (0.53)	3.78 (0.53)	2.48 (0.54)	3.62 (0.49)	*F* (3, 187) = 70.70, *p* < 0.001	4.06 × 10^+27^	0.53	0.53

*Note*. η_p_^2^ represents partial eta squared. ANCOVAs were performed with gender and age as covariates. Eta squared accounts for the effects of profiles without the effects of age and gender. Vovk-Sellke Maximum p-Ratio is reported using the p-value; this value provides the maximal odds in favour of H_1_ over H_0_ equals 1/(−e p log(p)) for *p* ≤ 0.37 (Sellke et al. 2001).

**Table A2:** Means, Standard Deviations, ANCOVA F statistic, Vovk-Sellke Maximum p-Ratio, and eta squared of AMPD-Only Profiles.

	Mean (SD) of High Psychopathology	Mean (SD) of Moderate Psychopathology	Mean (SD) of Low Psychopathology	F Statistic	VS-MPR	η^2^	η_p_^2^
Negative Affectivity	2.21 (0.43)	1.64 (0.49)	0.86 (0.41)	*F* (2, 188) = 64.08, *p* < 0.001	1.25 × 10^+19^	.39	.41
Detachment	1.67 (0.46)	1.38 (0.58)	0.79 (0.54)	*F* (2, 188) = 29.52, *p* < 0.001	2.02 × 10^+9^	0.23	0.24
Antagonism	1.23 (0.61)	0.67 (0.42)	0.33 (0.27)	*F* (2, 188) = 29.99, *p* < 0.001	2.85 × 10^+9^	0.23	0.24
Disinhibition	1.95 (0.31)	1.17 (0.36)	0.53 (0.24)	*F* (2, 188) = 143.94, *p* < 0.001	3.46 × 10^+35^	0.60	0.60
Psychoticism	1.61 (0.57)	0.86 (0.42)	0.31 (0.25)	F (2, 188) = 65.79, *p* < 0.001	3.36 × 10^+19^	0.41	0.41

*Note*. η_p_^2^ represents partial eta squared. ANCOVAs were performed with gender and age as covariates. Eta squared accounts for the effects of profiles without the effects of age and gender. Vovk-Sellke Maximum p-Ratio is reported using the p-value; this value provides the maximal odds in favour of H_1_ over H_0_ equals 1/(−e p log(p)) for *p* ≤ 0.37 (Sellke et al. 2001).

**Table A3:** Means, Standard Deviations, ANCOVA F statistic, Vovk-Sellke Maximum p-Ratio, and eta squared of FFM–AMPD Combined Profiles.

	Mean (SD) of Externalizing	Mean (SD) of Adaptive	Mean (SD) of Average-Withdrawn	Mean (SD) of Internalizing	F Statistic	VS-MPR	η^2^	η_p_^2^
Openness	3.89 (.46)	4.13 (.60)	3.69 (.58)	3.69 (.65)	*F* (3, 187) = 6.10, *p* < 0.001	88.35	.08	.09
Neuroticism	3.96 (.61)	3.03 (.76)	3.63 (.52)	4.36 (.34)	*F* (3, 187) = 34.50, *p* < 0.001	1.09	.33	.36
Agreeableness	3.59 (.57)	4.37 (.44)	4.09 (.48)	4.19 (.40)	*F* (3, 187) = 19.79, *p* < 0.001	4.39 × 10^+8^	.22	.24
Conscientiousness	2.81 (.57)	3.57 (.61)	3.02 (.54)	2.69 (.51)	*F* (3, 187) = 19.96, *p* < 0.001	5.25 × 10^+8^	.23	.24
Extraversion	3.30 (.49)	3.90 (.46)	2.80 (.68)	2.12 (.45)	*F* (3, 187) = 77.92, *p* < 0.001	5.17 × 10^+29^	.55	.56
Negative Affectivity	2.02 (.49)	1.25 (.66)	1.57 (.45)	2.26 (.38)	*F* (3, 187) = 26.16, *p* < 0.001	3.33 × 10^+11^	.27	.30
Detachment	1.47 (.44)	.65 (.35)	1.61 (.37)	2.06 (.42)	*F* (3, 187) = 96.40, *p* < 0.001	4.55 × 10^+34^	.60	.61
Antagonism	1.39 (.47)	.56 (.43)	.60 (.37)	.48 (.41)	*F* (3, 187) = 36.37, *p* < 0.001	6.14 × 10^+15^	.35	.37
Disinhibition	1.88 (.37)	.86 (.46)	1.07 (.35)	1.66 (.35)	*F* (3, 187) = 61.31, *p* < 0.001	4.96 × 10^+24^	.48	.50
Psychoticism	1.40 (.56)	.69 (.53)	.70 (.37)	1.58 (.59)	*F* (3, 187) = 30.48, *p* < 0.001	2.41 × 10^+13^	.31	.33

*Note*. η_p_^2^ represents partial eta squared. ANCOVAs were performed with gender and age as covariates. Eta squared accounts for the effects of profiles without the effects of age and gender. Vovk-Sellke Maximum p-Ratio is reported using the p-value; this value provides the maximal odds in favour of H_1_ over H_0_ equals 1/(−e p log(p)) for *p* ≤ 0.37 (Sellke et al. 2001).
